# Variants in the *CYP7B1* gene region do not affect natural resistance to HIV-1 infection

**DOI:** 10.1186/s12977-015-0206-0

**Published:** 2015-09-24

**Authors:** Manuela Sironi, Mara Biasin, Chiara Pontremoli, Rachele Cagliani, Irma Saulle, Daria Trabattoni, Francesca Vichi, Sergio Lo Caputo, Francesco Mazzotta, Wbeimar Aguilar-Jimenez, Maria Teresa Rugeles, Samandhy Cedeno, Jorge Sanchez, Christian Brander, Mario Clerici

**Affiliations:** Scientific Institute IRCCS E. MEDEA, Bioinformatics, 23842 Bosisio Parini, Italy; Department of Biomedical and Clinical Sciences “L. Sacco”, University of Milan, Via G.B. Grassi 74, 20157 Milan, Italy; S. Maria Annunziata Hospital, 50122 Florence, Italy; Immunovirology Group, School of Medicine, University of Antioquia UdeA, Medellín, Colombia; AIDS Research Institute-IrsiCaixa-HIVACAT, Hospital Germans Trias i Pujol, Universitat Autònoma de Barcelona, Badalona, Institució Catalana de Recerca i Estudis Avançats (ICREA), Barcelona, Spain; University of Vic and Central Catalonia, Vic, Spain; Asociación Civil Impacta Salud y Educación, Lima, Peru; Department of Physiopathology and Transplantation, University of Milan, 20090 Milan, Italy; Don C. Gnocchi Foundation ONLUS, IRCCS, 20148 Milan, Italy

**Keywords:** HIV, HESN, CYP7B1, Resistance to infection

## Abstract

**Background:**

The genetic bases of natural resistance to HIV-1 infection remain largely unknown. Recently, two genome-wide association studies suggested a role for variants within or in the vicinity of the *CYP7B1* gene in modulating HIV susceptibility. CYP7B1 is an appealing candidate for this due to its contribution to antiviral immune responses. We analyzed the frequency of two previously described *CYP7B1* variants (rs6996198 and rs10808739) in three independent cohorts of HIV-1 infected subjects and HIV-1 exposed seronegative individuals (HESN).

**Findings:**

rs6996198 and rs10808739 were genotyped in three case/control cohorts of sexually-exposed HESN and HIV-1-infected individuals from Italy, Peru and Colombia. Comparison of the allele and genotype frequencies of the two SNPs under different models showed that the only significant difference was seen for rs6996198 in the Peruvian sample (nominal p = 0.048, dominant model). For this variant, a random-effect meta-analysis yielded non-significant results (dominant model, p = 0.78) and revealed substantial heterogeneity among cohorts. No significant effect of the rs10808739 allelic status on HIV-1 infection susceptibility (additive model, p = 0.30) emerged from the meta-analysis.

**Conclusions:**

Although our study had limited power to detect association due to the small sample size, comparisons among the three cohorts revealed very similar allelic and genotypic frequencies in HESN and HIV-1 positive subjects. Overall, these data indicate that the two GWAS-defined variants in the *CYP7B1* region do not strongly influence HIV-1 infection susceptibility.

## Findings

Individual variation in susceptibility to HIV-1 infection is well documented [1–3], and a minority of individuals (15 % of the HIV exposed people), usually referred to as HIV-1 exposed seronegative individuals (HESN), does not become productively infected despite multiple exposures to the virus [2, 4, 5]. Because only a minority of these subjects is homozygous for the *CCR5*Δ*32* deletion [6], it is logical to postulate that other, additional genetic factors can modulate susceptibility to HIV-1 infection. Some of such factors were identified through candidate gene approaches; only few of them however were replicated across different samples [6]. In recent years, genome-wide association studies (GWAS) have been used for the identification of common variants that underlie complex phenotypes. Among these, two GWASs for HIV-1 infection susceptibility have suggested a role of variants within or in the vicinity of the *CYP7B1* gene in this phenomenon [7, 8]. In one study, HIV-1 infected individuals were compared with HIV-1 negative subjects [7]. Using meta-analysis of two European cohorts and further validation in European Americans, a single signal with genome-wide significance was detected at rs6996198, a SNP that is located downstream the *CYP7B1* gene and possibly modulates its expression [7]. Another variant in *CYP7B1* (rs10808739, in intron 1) was described in a GWAS of HIV-1 serodiscordant couples from Eastern and Southern Africa. Although the variant did not reach genome-wide significance, it was one of the top signals in the study [8].

The *CYP7B1* gene encodes an enzyme belonging to the cytochrome P450 superfamily that is involved in cholesterol catabolism by inactivation of oxysterols and their subsequent conversion into bile salts, mainly in the liver, brain, and reproductive tract [9]. Controlling intracellular cholesterol metabolism is essential for the correct entry, assembly and budding of virions, and many viruses are dependent on cellular lipid metabolisms to ensure the correct budding of functional virions from infected cells [10]. HIV alters cholesterol trafficking switching it from a physiological efflux to a virus-controlled transport, thus reducing the ability of a cell to export cholesterol. CYP7B1 also modulates several immune functions, as well including proinflammatory cytokine release [11] and, via its catabolite 25-hydroxycholesterol [12], programmed cell death [13] and the synthesis of IgA [14], a class of antibodies that has been associated with resistance to HIV infection [15]. Hence, CYP7B1 represents an appealing candidate as a modifier of infection susceptibility due to its contribution to the synthesis of virions and the initiation of antiviral defense.

To verify the role played by CYP7B1 in resistance to HIV infection we recruited 125 Italian HESN exposed through unprotected sexual intercourse (SexExp-HESN). Inclusion criteria were a history of multiple unprotected sexual episodes for more than 4 years at the time of enrolment, with at least three episodes of at-risk intercourse within 4 months prior to study entry and an average of 30 (range, 18 to >100) reported unprotected sexual contacts per year [5]. Infection in HESN was ruled-out by plasma HIV RNA and proviral DNA analyses. HESN and 114 seropositive (SP) partners were recruited at the S. M. Annunziata Hospital, Florence; all of them were Italian of Caucasian origin.

Sixty-two Colombian SexExp-HESN and 51 SP partners were also included. The inclusion criteria for these HESN subjects were previously reported [16] and included a negative HIV-1/2 ELISA test within 1 month of sample taking. The similar ancestry component and pair-wise fixation index (FST) values in the Colombian cohort [17] indicated no intra-cohort stratification by ethnicity.

The third HESN cohort was recruited in Peru and has been described [18, 19] both in terms of host genetics (HLA and KIR) and immune reactivity to HIV and viral co-pathogens. The similar frequency of HLA and KIR alleles [18] suggested no major intra-cohort stratification. For this study, samples were available for a total of 130 HESN and 95 SP individuals, all of whom were recruited at IMPACTA clinics across Lima. HESN were tested on a 3-monthly basis for newly acquired HIV infection. Risk criteria for the HESN cohort were more than five different sexual partners over the last 3 months, reported STI over the last 6 months, sexual intercourse with a known HIV SP partner in the last 6 months and having accepted money for sex as described previously [J. Coll et al. Bangkok AIDS Vaccine meeting 2011, Factors influencing recruitment, retention and seroconversion rates in MSM at high risk for HIV infection in Lima and Barcelona].

The study was designed and performed according to the Helsinki declaration (1975 revised in 2000) and was approved by the Ethics Committee of the participating units. All subjects provided written informed consent to participate in this study.

Genotyping of *CYP7B1* rs6996198 and rs10808739 was performed by TaqMan probe assays (TaqMan SNP genotyping assay, Applied Biosystems, Foster City, CA, USA) using the allelic discrimination real-time PCR method. Genotyping rate was >0.90 for both SNPs in all samples. The two SNPs complied to Hardy–Weinberg equilibrium in all samples with the only exception of rs6966198 in the SP Peruvian sample (uncorrected p value = 0.02). Association analysis for single variants was performed using logistic regressions under different models using the PLINK software [20]. Meta-analysis was performed using a random effect model using the R package “meta” (R package version 4.3-0. http://CRAN.R-project.org/package=meta). Linkage analysis was performed using HaploReg v2 [21].

The two *CYP7B1* SNPs previously suggested to be associated with HIV-1 infection susceptibility (rs6996198 and rs10808739) are located ~177 kb apart and are not in strong linkage disequilibrium (LD) in individuals of European or American descent (*r*^*2*^ = 0.15 and *r*^*2*^ = 0.12, respectively, http://analysistools.nci.nih.gov/LDlink/). The two variants were genotyped in three distinct cohorts of HESN and geographically matched HIV-1 SP subjects (Table 1). The main risk factor in all HESN was unprotected sex.

**Table 1:** Demographic status of the populations examined

Characteristics	Colombia		Italy		Peru	
	SP (n = 51)	HESN (n = 62)	SP (n = 114)	HESN (n = 125)	SP (n = 95)	HESN (n = 130)
Age, mean years ± SD	33.9 ± 7.5	35.1 ± 10.6	42.4 ± 8.8	41.6 ± 9.1	30.8 ± 6.7	31.2 ± 10.7
Males, n (%)	26 (50)	27 (44.2)	69 (60.5)	53 (42.4)	94 (99)	123 (90)
Viral load, median copies/mL (interquartile range)	2569 (488–25,075)	–	10,950 (395–27,410)	–	29,694 (11,162–63,381)	–
CD4+ T cell/µL count, median (interquartile range)	366 (190–568)	–	369 (239–554)	–	417^e^ (331–544)	–
Monthly unprotected sexual episodes, mean (range)^c^	8 (1–30)	8 (1–30)	3 (1.5–10)		7 (1–25)	
Previous history of sexually transmitted diseases and/or AIDS-defining illnesses (%)	40	22^a^	39	–	nd^d^	29
Heterosexual orientation (%)	79	90	100	100	nd^d^	17
Homosexual orientation (%)	3	2	0	0	nd^d^	44
Bisexual orientation (%)	17	7	0	0	nd^d^	39
Ethnicity, ancestry^b^ (%)	Afr: 25	Afr: 22	European (Tuscan): 100	European (Tuscan): 100	Mestizo: 100	Mestizo: 89
	Amer: 40	Amer: 42				Indigenous: 5
	Eur: 34	Eur: 35				Others: 6

*SP* seropositives, *HESN* HIV-1 exposed seronegative, *SD* standard deviation, *yrs* years, *Afr* African, *Amer* Amerindian, *Eur* European

^a^In HESNs sexually transmitted diseases but no AIDS-defining illnesses

^b^Ancestry of the Colombian cohort was previously reported [17]

^c^In Peru, this refers to number of partners, not sexual episodes

^d^“nd” is not determined. The majority is gay men but this question was not formally asked

^e^Cohort inclusion criteria was CD4 count >250 (requested by ethics board)

The allele and genotype frequencies at the two SNPs were compared under different models. The only significant difference was observed for rs6996198 in the Peruvian sample (nominal p = 0.048, dominant model): as in the original GWAS [7], the frequency of TT + CT genotypes was lower in SP compared to HESN subjects (Table 2). For this variant, a random-effect meta-analysis yielded non-significant results (dominant model, p = 0.78, OR = 1.0971, 95 % CI: 0.5727–2.1016) and revealed substantial heterogeneity among cohorts (Cochrane’s Q p value = 0.047, I^2^ = 67.32). Likewise, meta-analysis indicated no significant effect of rs10808739 allelic status on HIV-1 infection susceptibility (additive model, p = 0.30, OR = 0.85, 95 % CI: 0.6309–1.1531); no heterogeneity was observed for this variant (Cochrane’s Q p value = 0.700, I^2^ = 0).

**Table 2:** Association of rs6966198 and rs10808739 with HIV-1 infection susceptibility

Sample	Genotype frequency				Allelic frequency		*p*_additive_^b^	*p*_dominant_^c^	*p*_recessive_^d^
	CC	CT	TT	CT + TT^a^	C	T			
rs6966198									
Italian HESN	0.648	0.304	0.048	0.35	0.8	0.2	0.112	0.137	0.373
Italian SP	0.551	0.373	0.076	0.45	0.737	0.263			
Colombian HESN	0.661	0.274	0.065	0.339	0.798	0.202	0.557	0.83	0.309
Colombian SP	0.681	0.298	0.021	0.319	0.83	0.17			
Peruvian HESN	0.685	0.292	0.023	0.315	0.831	0.169	0.147	0.048	0.399
Peruvian SP	0.805	0.152	0.043	0.196	0.88	0.12			

Sample	Genotype frequency				Allelic frequency		*p*_additive_^b^	*p*_dominant_^c^	*p*_recessive_^d^
	GG	GA	AA	–	G	A			
rs10808739									
Italian HESN	0.58	0.33	0.09	–	0.746	0.254	0.392	0.267	0.961
Italian SP	0.5	0.412	0.088	–	0.706	0.294			
Colombian HESN	0.71	0.226	0.064	–	0.823	0.177	0.831	0.787	0.275
Colombian SP	0.686	0.294	0.02	–	0.833	0.167			
Peruvian HESN	0.794	0.198	0.008	–	0.893	0.107	0.302	0.38	0.389
Peruvian SP	0.741	0.235	0.024	–	0.859	0.141			

^a^Genotypic counts (dominant model, as in [7])

^b^Logistic regression p value for an additive model

^c^Logistic regression p value for a dominant model

^d^Logistic regression p value for a recessive model

**Fig. 1:**
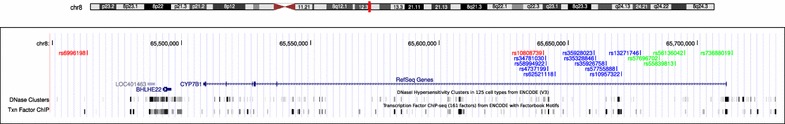
Analyzed variants and LD analysis. Representation of the CYP7B1 gene region within the UCSC Genome Browser view. The two variants we genotyped are shown in *red*. Variants in LD (*r* ^*2*^ ≥ 0.8) with rs10808739 in Africans, Americans, and Europeans are reported in *blue*. SNPs in *green* show LD (*r* ^*2*^ ≥ 0.8) with rs10808739 in African populations only. Relevant ENCODE regulation tracks are shown

The original study that reported association of rs6996198 with protection from HIV-1 acquisition was performed in European populations; analysis of the 1000 Genomes Project data (Phase I) indicated that no variant in strong LD (*r*^*2*^ ≥ 0.8) with rs6996198 segregates in Europeans (nor in Americans). As for rs10808739, it was initially described in cohorts of African ancestry. LD analysis in African populations indicated that rs10808739 is in strong LD (*r*^*2*^ ≥ 0.8) with several variants, the majority of which show a similar LD pattern in Europeans and Americans (Fig. 1). Thus, it seems unlikely that the negative results we obtained are due to substantially different haplotype structures across populations.

CYP7B1 plays a major role in the synthesis of HIV and in immunity and is thus a likely candidate in the modulation of susceptibility to HIV. Results, however, did not confirm the previously reported associations, suggesting that these particular *CYP7B1* genetic variants do not play a role in HIV-1 infection susceptibility, and indicating that other variants will need to be analyzed. A limitation of our study is the relatively small sample sizes of the HESN and HIV-1 infected cohorts, resulting in low power to detect potentially existing associations. Nonetheless, the combined sample size was comparable to that analyzed in the serodiscordant couple study that detected an association at rs10808739 [8]. As for rs6966198, it reached genome-wide significance in a relatively large two-stage study that employed a different design from the one applied herein [7]. Thus, in that case, HIV-1-infected individuals were compared to HIV-1 negative subjects, irrespective of their exposure status. This may have determined a proportion of HIV-1 negative controls to be misclassified—i.e. most of with a consequent reduction of power. Our analyses were performed in well characterized HESN cohorts hence overcoming this problem; a degree of misclassification may nevertheless still remain for HIV-1 positive subjects because their exposure history may not always be properly and completely recorded and their group is likely to include individuals in a wide susceptibility range.

Taking into account these limitations, comparisons in the three cohorts showed very similar allelic and genotypic frequencies in HESN and HIV-1-infected individuals, with the only exception being the Peruvian sample that reached nominal significance for rs6996198. For the same variant, the Italian cohort also showed some difference in frequency, albeit not significant, that was in the opposite direction to that reported by the original association study [7] and to that observed in Peruvians. The original study that identified rs6996198 as potentially associated with HIV-1 infection susceptibility estimated an OR of 0.64 with narrow confidence intervals (95 % IC: 0.54–075). Herein, also due to heterogeneity among samples, we obtained an OR very close to 1 with wide uncertainty (95 % IC: 0.5727–2.1016). Thus, our results do not imply that rs6996198 has no role in modulating HIV-1 infection susceptibility, but indicate that its effect (if any) is small. Overall, these data lead us to conclude that the two genotyped variants in the *CYP7B1* region do not strongly influence HIV-1 infection susceptibility.
